# Machine learning to develop and validate a model for predicting the risk of lymph node metastasis in colorectal cancer patients

**DOI:** 10.3389/fonc.2026.1860520

**Published:** 2026-07-21

**Authors:** Changhe Xia, Fang Liu, Changjiang Xia, Yimin Wang, Xiaohong Zheng, Zhanxue Zhang, Feifei Wang, Chaoxi Zhou, Guiying Wang

**Affiliations:** 1 Department of Proctology, Qinhuangdao First Hospital, Qinhuangdao, Hebei, China; 2 Department of Physical Examination Center, Qinhuangdao First Hospital, Qinhuangdao, Hebei, China; 3 Department of Pharmacy, Tangshan People’s Hospital, Tangshan, Hebei, China; 4 Department of General Surgery, Qinhuangdao First Hospital, Qinhuangdao, Hebei, China; 5 Department of Gastrointestinal Surgery, The Second Hospital of Hebei Medical University, Shijiazhuang, Hebei, China; 6 The Second Department of General Surgery, The Fourth Hospital of Hebei Medical University, Shijiazhuang, Hebei, China; 7 Department of General Surgery, The Second Hospital of Hebei Medical University, Shijiazhuang, Hebei, China; 8 Hebei Key Laboratory of Etiology Tracing and Individualized Diagnosis and Treatment for Digestive system carcinoma, The Second Hospital of Hebei Medical University, Shijiazhuang, Hebei, China

**Keywords:** colorectal cancer, lymph node metastasis, machine learning, model construction and validation, predictive model

## Abstract

**Background:**

The presence of lymph node metastasis (LNM) serves as a critical determinant of prognosis in colorectal cancer (CRC), often correlating with markedly unfavorable clinical outcomes. Consequently, this investigation sought to construct and rigorously validate a machine learning (ML) algorithm capable of estimating LNM probability within the CRC population.

**Methods:**

Through the application of logistic regression analysis, six independent predictors were identified: extramural vascular invasion (EMVI), T stage, fibrinogen (FIB), systolic blood pressure (SBP), thrombin time (TT), and alpha-fucosidase (AFU). Subsequently, diverse ML architectures were generated and assessed utilizing receiver operating characteristic (ROC) curves, calibration plots, and decision curve analysis (DCA).

**Results:**

Among the evaluated algorithms, the stochastic gradient boosting (gbm) model exhibited superior discriminatory power, yielding area under the curve (AUC) metrics of 0.815 in the training cohort and 0.733 in the external validation set. Calibration assessments revealed a robust concordance between projected probabilities and actual observed events. Notably, the xgbTree algorithm also demonstrated commendable predictive efficacy.

**Conclusion:**

We successfully established and verified an ML-based framework designed to forecast LNM risk in individuals with CRC. These computational tools offer clinicians a refined mechanism for the precise identification of nodal involvement, thereby laying the groundwork for formulating personalized therapeutic regimens.

## Highlights

- Machine learning model predicts lymph node metastasis in colorectal cancer pre-surgery.
- Key predictors include EMVI, T stage, fibrinogen, systolic blood pressure, TT, and AFU.
- The GBM algorithm shows robust performance in both internal and external validation.
- Model may aid in personalized treatment planning, like neoadjuvant therapy decisions.
- Study validates a non-invasive tool using readily available clinical variables.

## 1 Introduction

Colorectal cancer (CRC) represents the third most frequently diagnosed malignancy globally and is the second primary cause of cancer-associated mortality (1–3). Epidemiological data indicate that CRC contributes to over 1.85 million new diagnoses and approximately 850,000 fatalities annually (4). The disparity in five-year survival rates between patients presenting with stage I versus stage IV disease is profound, underscoring the vital necessity for early diagnostic intervention (5, 6). Currently, clinical management protocols for CRC rely heavily on the tumor-node-metastasis (TNM) classification system endorsed by the American Joint Committee on Cancer (AJCC) (7). The status of lymph node metastasis exerts a pivotal influence on therapeutic decision-making for CRC patients, particularly regarding the administration of preoperative neoadjuvant chemotherapy (8). Chemo radiotherapy (CRT) combined with surgical intervention remains a cornerstone of treatment (9). Furthermore, precise determination of lymph node metastasis (LNM) status is instrumental in guiding therapeutic strategies for early-stage colorectal cancer (CRC), particularly regarding the choice between endoscopic resection and radical surgery (10). Beyond treatment selection, LNM status serves as a critical prognostic indicator for both overall survival (OS) and disease-free survival (DFS) in patients with M0-stage disease (11, 12).

Numerous investigations have sought to establish predictive frameworks for identifying LNM risk factors. While computed tomography (CT) constitutes a fundamental component of CRC diagnostic workups and offers some capacity to detect nodal involvement (13), its sensitivity and specificity remain suboptimal for distinguishing malignant from benign nodes during local staging (14). Conversely, integrating serum angiopoietin-like protein 2 levels with clinical parameters has demonstrated superior accuracy in preoperatively predicting CRC lymph node metastasis (15). Additionally, Ueno et al. identified poor tumor differentiation, definite vascular invasion, and tumor budding as independent predictors of LNM (16). Although various methodologies and biomarkers have been explored to forecast LNM status (17, 18), a significant gap persists: there is currently a lack of robust, reliable tools for LNM prediction, underscoring an urgent need for novel diagnostic solutions.

Machine learning (ML), an emerging artificial intelligence paradigm, has exhibited remarkable accuracy and generalizability in diagnosing malignant tumors and predicting patient prognosis (19–21). Its application within medical data analytics has become increasingly widespread (22, 23). Distinct from conventional statistical approaches, ML algorithms excel at uncovering vital predictors closely linked to outcome metrics by modeling complex variable interactions. By learning patterns inherent in datasets, ML identifies optimized associations between target outcomes and potential predictors, thereby enhancing model precision (24, 25). Despite these advantages, few studies have leveraged ML algorithms to evaluate LNM risk specifically in CRC populations.

Consequently, this study aimed to develop and validate a machine learning-based model for predicting LNM risk in CRC patients using a multicenter cohort. We constructed the model using data extracted from a primary center and performed external validation utilizing datasets from independent institutions. The resulting tool is designed to assist clinicians in formulating personalized therapeutic decisions.

## 2 Methods

### 2.1 Study cohort

**Figure 1:**
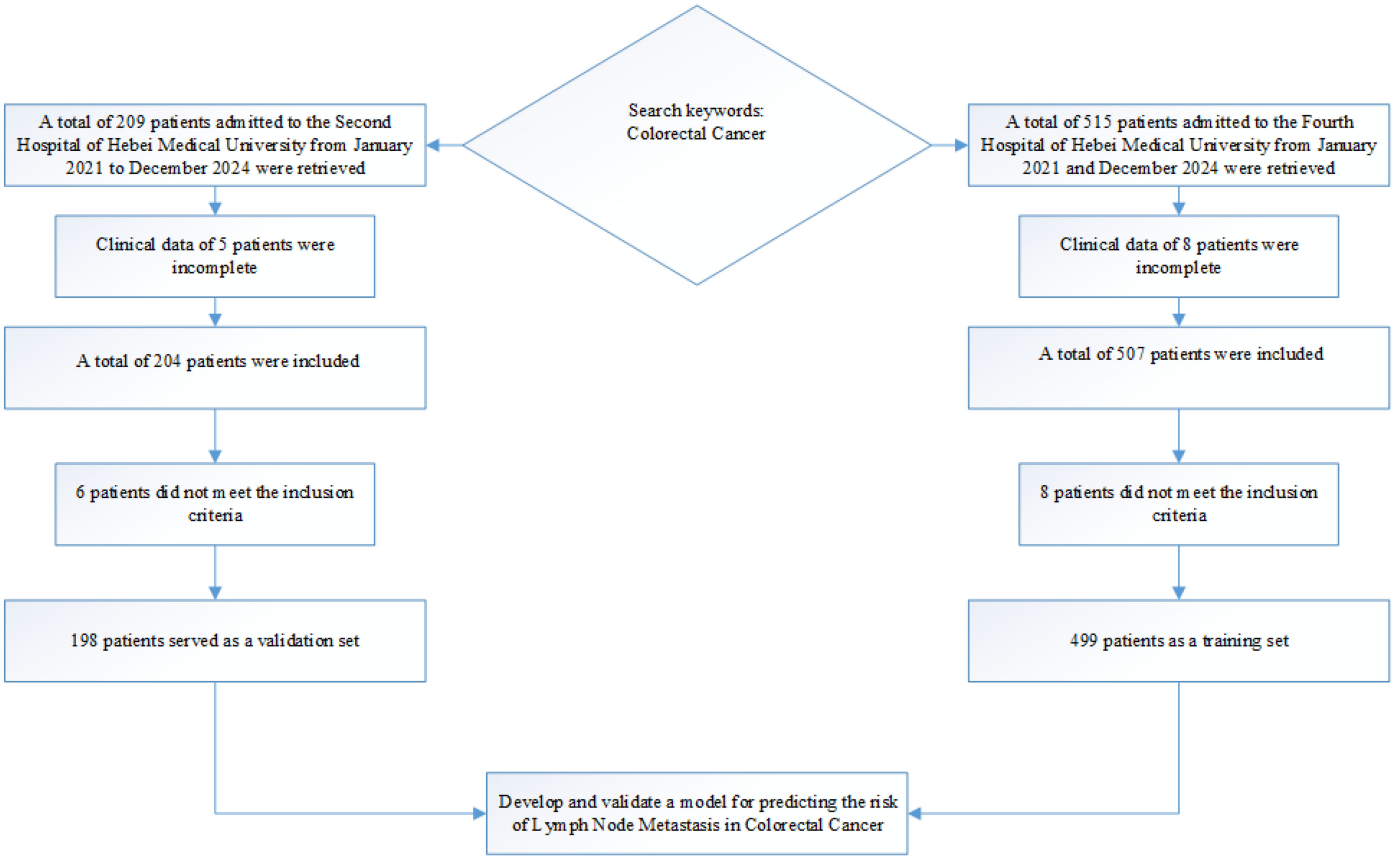
The flowchart of this study.

We retrospectively collected data from CRC patients admitted to the Fourth Hospital and the Second Hospital of Hebei Medical University between January 2021 and December 2024. To ensure accurate pathological assessment of LNM status, inclusion was restricted to patients who underwent surgical resection with complete specimen availability. The specific inclusion criteria were as follows: (1) availability of histological sections and comprehensive clinicopathological data for the primary tumor; (2) absence of synchronous or metachronous malignancies; and (3) no history of hereditary CRC syndromes. The exclusion criteria were as follows: (1) incomplete or missing clinical records; (2) unsatisfactory quality of histological sections; (3) performance of emergency surgery; and (4) presence of distant metastases. Ultimately, data from 499 patients at the Fourth Hospital served as the training set, while data from 198 patients at the Second Hospital constituted the external validation cohort. This study protocol adhered to the Declaration of Helsinki guidelines and received approval from the Ethics Committees of both the Fourth and Second Hospitals of Hebei Medical University. Waiver of informed consent: The Ethics Committees of both the Fourth and Second Hospitals affiliated with Hebei Medical University granted a waiver for informed consent requirements. **Figure 1** illustrates the overall study workflow.

### 2.2 Clinicopathological features

Comprehensive data were extracted from electronic health records, encompassing baseline demographics, primary tumor T staging (per AJCC 8th Edition), laboratory profiles, and postoperative pathological findings. Specifically, the dataset included tumor location, sex, age, body mass index (BMI), pulse rate, systolic blood pressure (SBP), diastolic blood pressure (DBP), percentages of neutrophils, lymphocytes, and monocytes, hemoglobin levels, platelet counts, AST/ALT ratios, prealbumin (PAB), total protein (TP), albumin (ALB), globulin (GLB), alkaline phosphatase (ALP), γ-glutamyl trans peptidase (GGT), adenosine deaminase (ADA), α-L-fucosidase (AFU), glucose (GLU), prothrombin time (PT), prothrombin activity (PTA), thrombin time (TT), fibrinogen (FIB), β2-microglobulin, carcinoembryonic antigen (CEA), ferritin, carbohydrate antigen 19-9 (CA19-9), carbohydrate antigen 72-4 (CA72-4), TNM stage, tumor volume (TV), tumor budding grade, extramural vascular invasion (EMVI), nerve invasion (NI), and neoadjuvant chemotherapy (NAC) status.

Definitions of key variables. Extramural vascular invasion (EMVI) was defined as the presence of a definite tumor signal within a vascular structure adjacent to the primary tumor on preoperative T2−weighted and contrast−enhanced MRI sequences, following the criteria established by Smith et al (26). Clinical T stage was determined according to the AJCC Cancer Staging Manual (8th edition) (27) based on preoperative contrast−enhanced CT or MRI: T1 indicated tumor invasion of the submucosa; T2 indicated invasion of the muscularis propria; T3 indicated invasion through the muscularis propria into the pericolic or perirectal tissues; and T4 indicated direct invasion of other organs or structures (T4a) or perforation of the visceral peritoneum (T4b). Tumor volume (TV) was measured on axial CT or MRI images and calculated using the ellipsoid formula (28): volume = length × width × height × π/6, where length, width, and height represent the maximal tumor dimensions in three orthogonal planes. All measurements were performed independently by two senior radiologists blinded to the patients’ clinical outcomes, and the average value was used for analysis.

### 2.3 Data processing

Categorical variables underwent one-hot encoding, whereas all laboratory metrics were retained as continuous values without discretization. These preprocessing steps were applied uniformly to both the training and testing cohorts. No missing data were present in the final analytical dataset because patients with incomplete or missing clinical records were excluded according to the exclusion criteria described in section 2.1. Therefore, no imputation methods were applied.

### 2.4 Selection of variables

To mitigate overfitting in multifactorial models, we employed multivariate logistic regression for feature screening within the training cohort. Only variables demonstrating statistical significance (P < 0.05) were subsequently integrated into the machine learning frameworks. All six independent predictors included in the final model were obtained from preoperative assessments. Clinical T stage and extramural vascular invasion (EMVI) were determined using preoperative contrast−enhanced CT or MRI according to the AJCC 8th Edition criteria and published radiological definitions, respectively. Fibrinogen, thrombin time, alpha−fucosidase, and systolic blood pressure were collected from routine preoperative blood tests and vital sign measurements.

### 2.5 Model construction and validation

Eight distinct machine learning algorithms—logistic regression (LR), decision tree (DT), random forest (RF), K-nearest neighbors (KNN), naive Bayes (NB), extreme gradient boosting (XGB), stochastic gradient boosting (SGBoost), and neural networks (NNET)—were implemented to predict lymph node metastasis risk in colorectal cancer. We utilized tenfold cross-validation to optimize model performance. Optimal hyper parameters for each algorithm were identified through default grid search procedures within the “caret” package (R software) using the selected feature subset. Consequently, the final models were constructed on the training set utilizing these optimized parameters and features. We implemented eight machine learning algorithms to predict lymph node metastasis risk. Briefly, logistic regression (LR) models the log−odds of the outcome as a linear combination of predictors; decision tree (DT) partitions the feature space recursively based on simple decision rules; random forest (RF) averages many decorrelated decision trees to reduce variance; K−nearest neighbors (KNN) classifies a sample based on the majority class among its closest neighbors in the feature space; naive Bayes (NB) applies Bayes’ theorem with strong independence assumptions between features; extreme gradient boosting (xgbTree) and stochastic gradient boosting (gbm) build an ensemble of weak learners sequentially, each correcting the errors of its predecessor; and neural networks (nnet) use interconnected layers of nodes to model complex nonlinear relationships. For each algorithm, hyperparameters were optimized using ten−fold cross−validation with default grid search via the caret package in R. Hyperparameters for each machine learning algorithm were optimized using ten−fold cross−validation repeated once (or as you actually did) with the caret package (version 6.0−94) in R software (version 4.3.1). The tuning grids were defined using default ranges provided by caret for each method, with grid search applied to identify the combination that maximized the area under the receiver operating characteristic curve (AUC). The final optimal hyperparameters for each algorithm are summarized in **Supplementary Table 1**.

### 2.6 Model performance test

Model reliability was assessed using multiple metrics: area under the receiver operating characteristic curve (AUC), sensitivity, specificity, positive predictive value (PPV), negative predictive value (NPV), F1 score, balanced accuracy, and Brier score. Calibration curves were used to evaluate the agreement between predicted probabilities and observed outcomes. Furthermore, decision curve analysis (DCA) quantified the net clinical benefit across various threshold probabilities. The superior machine learning model was ultimately selected based on this comprehensive performance assessment.

To evaluate the robustness of the developed machine learning models, we adopted multiple strategies. First, ten−fold cross−validation repeated for ten iterations was used to reduce overfitting and assess the stability of model performance (29–31). Second, external validation on an independent cohort (Second Hospital of Hebei Medical University) was performed to test generalizability across different patient populations (32). Third, calibration curves were plotted to evaluate the agreement between predicted probabilities and observed outcomes, and Brier scores were calculated as a measure of overall model accuracy (32). Fourth, decision curve analysis (DCA) was conducted to quantify the net clinical benefit across a range of threshold probabilities, thereby assessing the practical utility of the models under different risk scenarios (33). These combined approaches provided a comprehensive evaluation of model robustness beyond simple discrimination metrics.

### 2.7 Statistics

All machine learning models were developed using R software (version 4.3.1) and the “caret” package. Implementation leveraged specific functions within “caret”: LR (glm), DT (rpart), RF (rf), KNN (knn), NB (nb), XGB (xgbTree), and SGBoost (by corresponding method parameters).

GBM, and NNET. Continuous variables adhering to a normal distribution are reported as the means ± standard deviations and were analyzed using t tests. For continuous variables exhibiting skewed distributions, data are presented as medians with interquartile ranges and compared via the Mann–Whitney U test or Kruskal–Wallis H test. Furthermore, categorical variables are summarized as counts and percentages, with comparisons conducted using chi-square tests or Fisher’s exact test. To identify independent risk factors within the training cohort, multivariate logistic regression was employed. Statistical significance was defined as a two-tailed P value < 0.05.

## 3 Results

### 3.1 Baseline data

The training cohort comprised 499 patients recruited from the Fourth Hospital of Hebei Medical University, among whom 186 (37.3%) exhibited lymph node metastasis (LNM). An external validation set consisting of 198 patients was sourced from the Second Hospital of Hebei Medical University; LNM was observed in 77 (38.9%) of these individuals. **Table 1** details the demographic and clinicopathological profiles for both cohorts. Variable distributions were generally balanced between the training and external testing sets. Notably, significant disparities emerged when comparing the non-LNM and LNM subgroups. Specifically, the LNM group demonstrated elevated levels of CEA (20.5 vs. 11.3, p = 0.0373) and CA19-9 (47.1 vs. 24.5, p = 0.0119), along with more advanced T stages (p < 0.001). Additionally, higher rates of extramural venous invasion (EMVI) positivity (30.8% vs. 4.4%, p < 0.001) and nerve invasion (NI) (28.5% vs. 11.1%, p < 0.001) were observed in the LNM cohort, whereas TT levels were significantly lower (14.0 vs. 14.6, p = 0.0385).

**Table 1:** The demographic and clinicopathological characteristics of patients in the training and testing sets.

Variable name	Test set	Train set	P value	Overall	N0	N1	P value
	(N = 198)	(N = 499)		(N = 697)	(N = 434)	(N = 263)	
Sex							
Female	85 (42.9%)	283 (56.7%)	0.00136	368 (52.8%)	226 (52.1%)	142 (54.0%)	0.886
Male	113 (57.1%)	216 (43.3%)		329 (47.2%)	208 (47.9%)	121 (46.0%)	
Age							
Mean ± SD	64.2 ± 11.7	64.1 ± 11.2	0.881	64.1 ± 11.4	64.4 ± 11.0	63.7 ± 11.9	0.717
beta2_MG							
Mean ± SD	2.58 ± 1.37	2.19 ± 6.74	0.224	2.30 ± 5.75	2.08 ± 0.733	2.67 ± 9.31	0.349
CEA							
Mean ± SD	15.7 ± 53.1	14.5 ± 39.4	0.774	14.8 ± 43.7	11.3 ± 27.2	20.5 ± 61.5	0.0373
CA19-9							
Mean ± SD	22.7 ± 46.4	37.2 ± 104	0.0116	33.1 ± 91.9	24.5 ± 66.0	47.1 ± 122	0.0119
T stage							
1	8 (4.0%)	31 (6.2%)	0.0183	39 (5.6%)	38 (8.8%)	1 (0.4%)	< 0.001
2	11 (5.6%)	61 (12.2%)		72 (10.3%)	59 (13.6%)	13 (4.9%)	
3	39 (19.7%)	72 (14.4%)		111 (15.9%)	67 (15.4%)	44 (16.7%)	
4	140 (70.7%)	335 (67.1%)		475 (68.1%)	270 (62.2%)	205 (77.9%)	
TV							
Mean ± SD	44.3 ± 77.2	38.8 ± 74.1	0.39	40.3 ± 74.9	40.2 ± 72.3	40.5 ± 79.2	0.999
EMVI							
No	157 (79.3%)	440 (88.2%)	0.00376	597 (85.7%)	415 (95.6%)	182 (69.2%)	< 0.001
Yes	41 (20.7%)	59 (11.8%)		100 (14.3%)	19 (4.4%)	81 (30.8%)	
NI							
No	153 (77.3%)	421 (84.4%)	0.0352	574 (82.4%)	386 (88.9%)	188 (71.5%)	< 0.001
Yes	45 (22.7%)	78 (15.6%)		123 (17.6%)	48 (11.1%)	75 (28.5%)	
NAC							
No	197 (99.5%)	447 (89.6%)	< 0.001	644 (92.4%)	395 (91.0%)	249 (94.7%)	0.209
Yes	1 (0.5%)	52 (10.4%)		53 (7.6%)	39 (9.0%)	14 (5.3%)	
BMI							
Mean ± SD	23.8 ± 3.46	24.1 ± 3.93	0.38	24.0 ± 3.80	23.9 ± 3.73	24.3 ± 3.91	0.313
Pulse							
Mean ± SD	84.4 ± 14.6	79.5 ± 12.5	< 0.001	80.9 ± 13.3	80.4 ± 13.5	81.7 ± 12.9	0.441
SBP							
Mean ± SD	126 ± 18.7	128 ± 18.1	0.229	128 ± 18.3	127 ± 18.1	129 ± 18.7	0.558
DBP							
Mean ± SD	81.4 ± 11.9	79.7 ± 9.81	0.0741	80.2 ± 10.5	80.1 ± 10.3	80.3 ± 10.7	0.952
neutrophil percentage							
Mean ± SD	64.7 ± 12.2	65.3 ± 10.6	0.537	65.1 ± 11.1	65.1 ± 11.6	65.2 ± 10.3	0.989
lymphocyte percentage							
Mean ± SD	25.4 ± 10.2	25.6 ± 9.56	0.823	25.5 ± 9.74	25.5 ± 10.1	25.5 ± 9.09	0.999
monocytes percentage							
Mean ± SD	7.73 ± 4.62	6.40 ± 2.23	< 0.001	6.78 ± 3.16	6.70 ± 3.39	6.91 ± 2.73	0.665
hemoglobin							
Mean ± SD	119 ± 24.8	122 ± 60.2	0.482	121 ± 52.6	120 ± 22.6	123 ± 80.6	0.785
Platelet count							
Mean ± SD	271 ± 105	273 ± 100	0.823	273 ± 101	270 ± 97.6	277 ± 107	0.645
AST/ALT							
Mean ± SD	1.44 ± 0.716	1.32 ± 0.493	0.0345	1.36 ± 0.567	1.36 ± 0.540	1.35 ± 0.611	0.999
PAB							
Mean ± SD	201 ± 66.1	208 ± 61.1	0.181	206 ± 62.6	208 ± 64.0	202 ± 60.2	0.422
TP							
Mean ± SD	67.9 ± 39.4	67.7 ± 23.9	0.946	67.7 ± 29.2	68.7 ± 36.6	66.2 ± 5.96	0.172
ALB							
Mean ± SD	40.2 ± 24.2	39.9 ± 4.64	0.856	40.0 ± 13.5	39.5 ± 5.07	40.8 ± 21.0	0.487
GLB							
Mean ± SD	27.6 ± 19.5	27.4 ± 12.5	0.854	27.5 ± 14.8	27.3 ± 13.5	27.8 ± 16.8	0.913
ALP							
Mean ± SD	83.2 ± 28.8	84.6 ± 24.7	0.551	84.2 ± 25.9	84.6 ± 23.4	83.5 ± 29.7	0.891
R_GT							
Mean ± SD	25.8 ± 28.7	25.5 ± 27.7	0.893	25.6 ± 28.0	24.1 ± 20.0	28.1 ± 37.5	0.214
ADA							
Mean ± SD	10.6 ± 3.80	11.3 ± 4.24	0.0208	11.1 ± 4.14	11.2 ± 4.16	11.0 ± 4.10	0.769
AFU							
Mean ± SD	15.9 ± 6.20	20.8 ± 12.7	< 0.001	19.4 ± 11.5	19.8 ± 13.3	18.8 ± 7.42	0.405
GLU							
Mean ± SD	7.63 ± 28.9	7.07 ± 31.7	0.821	7.23 ± 30.9	7.28 ± 33.9	7.14 ± 25.1	0.998
PT							
Mean ± SD	11.6 ± 1.86	11.7 ± 6.54	0.633	11.7 ± 5.62	11.6 ± 5.44	11.8 ± 5.91	0.908
PTA							
Mean ± SD	98.6 ± 55.1	101 ± 47.9	0.645	100 ± 50.0	102 ± 62.4	97.0 ± 14.0	0.118
TT							
Mean ± SD	14.7 ± 6.72	14.2 ± 4.19	0.363	14.4 ± 5.04	14.6 ± 6.22	14.0 ± 1.79	0.0385
FIB							
Mean ± SD	4.02 ± 5.65	3.54 ± 1.56	0.243	3.67 ± 3.29	3.60 ± 1.66	3.80 ± 4.92	0.753

Note: beta2_MG (β2-microglobulin), CEA (carcinoembryonic antigen), CA19-9 (carbohydrate antigen 19-9), TV (tumor volume), EMVI (extramural vascular invasion), NI (nerve invasion), NAC (neoadjuvant chemotherapy), BMI (body mass index), SBP (systolic blood pressure), DBP (diastolic blood pressure), PAB (prealbumin), TP (total protein), ALB (albumin), GLB (globulin), ALP (alkaline phosphatase), R_GT (R-glutamyl transpeptidase), ADA (adenosine deaminase), AFU (A-fucosidase), GLU (glucose), PT (prothrombin time), PTA (prothrombin activity), TT (thrombin time), FIB (fibrinogen).

### 3.2 Multivariable logistic regression analysis

Multivariate logistic regression was applied to the training cohort to delineate independent predictors of LNM. This analysis identified six variables independently associated with LNM risk in colorectal cancer patients: EMVI, clinical T stage, fibrinogen (FIB), systolic blood pressure (SBP), TT, and alpha-fucosidase (AFU) (**Table 2**).

**Table 2:** Multivariate logistic regression analysis was used to explore the independent risk factors for LNM in the training cohort.

Variable name	coef	std err	z	P > |z|	OR	OR_lower	OR_upper
EMVI	1.8757	0.666	2.816	0.005	6.525385	1.768267	24.07081
T stage	-0.7887	0.312	-2.528	0.011	0.454435	0.246597	0.83778
FIB	-0.8354	0.344	-2.428	0.015	0.433701	0.22091	0.851292
SBP	0.0358	0.015	2.41	0.016	1.036449	1.007025	1.067159
TT	-0.5277	0.229	-2.3	0.021	0.58996	0.376439	0.924964
AFU	-0.0559	0.028	-2.02	0.043	0.945634	0.895834	0.998002
GLU	-0.2228	0.113	-1.964	0.05	0.800275	0.640824	1
DBP	-0.0532	0.028	-1.892	0.058	0.94819	0.897628	1.002002
NAC	-1.2979	0.788	-1.647	0.099	0.273105	0.058309	1.2789
Monocytes percentage	0.1392	0.098	1.419	0.156	1.149354	0.94838	1.39236
TV	-0.0041	0.003	-1.412	0.158	0.995908	0.99005	1.002002
beta2_MG	-0.0273	0.02	-1.392	0.164	0.973069	0.936131	1.011061
AST_ALT	-0.4859	0.424	-1.145	0.252	0.615143	0.26767	1.413403
R_GT	-0.0069	0.007	-1.049	0.294	0.993124	0.980199	1.006018
CEA	-0.0051	0.005	-0.991	0.322	0.994913	0.985112	1.005013
CA19-9	-0.0018	0.002	-0.987	0.323	0.998202	0.995012	1.002002
Platelet count	0.0019	0.002	0.855	0.393	1.001902	0.998002	1.006018
Pulse	0.0083	0.016	0.531	0.596	1.008335	0.97824	1.03977
PAB	-0.0022	0.004	-0.515	0.607	0.997802	0.98906	1.006018
Hb	0.0039	0.008	0.496	0.62	1.003908	0.988072	1.020201
ALB	0.0287	0.063	0.455	0.649	1.029116	0.909373	1.165325
BMI	-0.0206	0.051	-0.405	0.685	0.979611	0.88692	1.082204
AGE	0.0076	0.02	0.381	0.703	1.007629	0.969476	1.047074
TP	-0.0178	0.053	-0.334	0.739	0.982357	0.885148	1.090897
NI	-0.1495	0.526	-0.284	0.776	0.861138	0.307279	2.413312
Neutrophil percentage	-0.0129	0.046	-0.281	0.778	0.987183	0.902127	1.080042
GLB	0.0076	0.027	0.277	0.781	1.007629	0.955042	1.062899
ADA	0.013	0.058	0.224	0.822	1.013085	0.903933	1.135417
Lymphocyte percentage	-0.0116	0.053	-0.22	0.826	0.988467	0.891366	1.096365
ALP	0.001	0.008	0.123	0.902	1.001001	0.984127	1.018163
PT	0.0025	0.025	0.103	0.918	1.002503	0.955042	1.052323
PTA	8.00E-04	0.009	0.097	0.923	1.0008	0.984127	1.018163
SEX	0.0285	0.42	0.068	0.946	1.02891	0.451581	2.344331

Note: beta2_MG (β2-microglobulin), CEA (carcinoembryonic antigen), CA19-9 (carbohydrate antigen 19-9), TV (tumor volume), EMVI (extramural vascular invasion), NI (nerve invasion), NAC (neoadjuvant chemotherapy), BMI (body mass index), SBP (systolic blood pressure), DBP (diastolic blood pressure), PAB (prealbumin), TP (total protein), ALB (albumin), GLB (globulin), ALP (alkaline phosphatase), R_GT (R-glutamyl transpeptidase), ADA (adenosine deaminase), AFU (A-fucosidase), GLU (glucose), PT (prothrombin time), PTA (prothrombin activity), TT (thrombin time), FIB (fibrinogen).

### 3.3 Model construction, validation, and performance test

**Figure 2:**
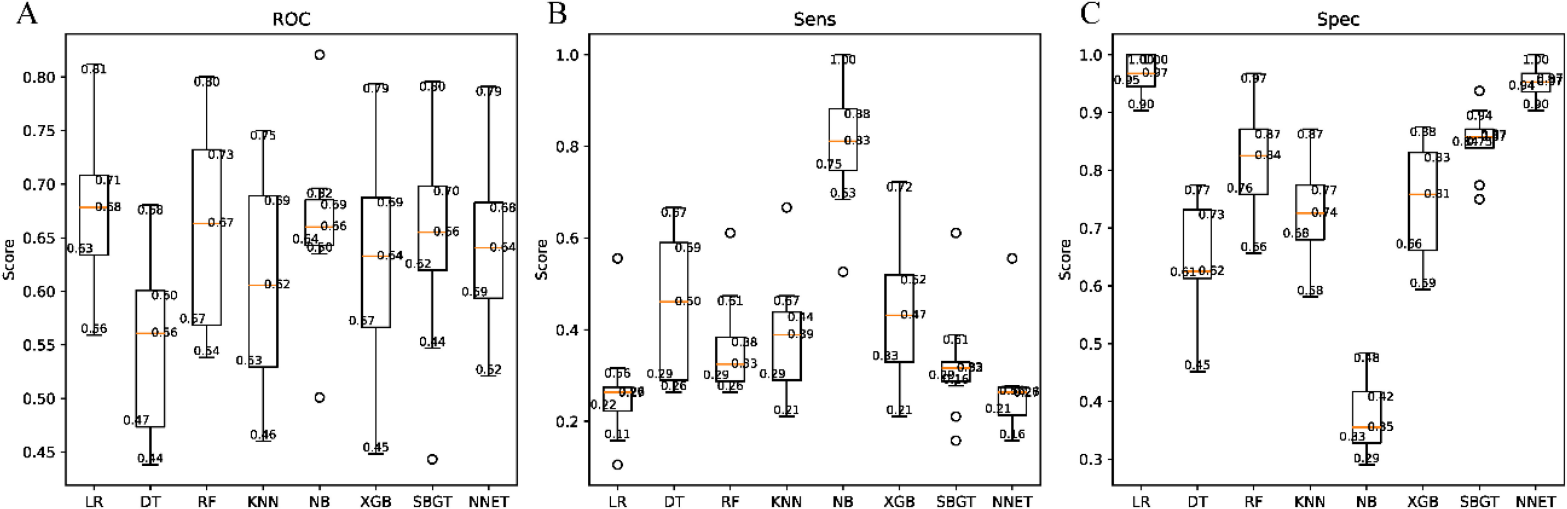
The internal validation results of the machine learning (ML) models. It displays the area under the curve (AUC) of receiver operating characteristic (ROC) curves, sensitivity (Sens), and specificity (Spec) of the ML models in internal validation. **(A)** AUC, **(B)** sensitivity, and **(C)** specificity. LR (logistic regression), DT (decision tree), RF (random forest), NB (naive Bayes), KNN (K-nearest neighbor), XGB (extreme gradient boosting), SBGT (stochastic gradient boosting), and NNET (neural network).

Eight machine learning (ML) algorithms were developed utilizing 10-fold cross-validation repeated across 10 iterations. Internal validation outcomes are illustrated in **Figure 1**. Regarding the area under the curve (AUC), the logistic regression (LR) model achieved superior performance (0.68, 95% CI: 0.621–0.737; **Figure 2A**). The naive Bayes (NB) model yielded the highest sensitivity (0.83, 95% CI: 0.706–0.900; **Figure 2B**), while the LR model demonstrated optimal specificity (0.968, 95% CI: 0.944–0.992; **Figure 2C**).

**Figure 3:**
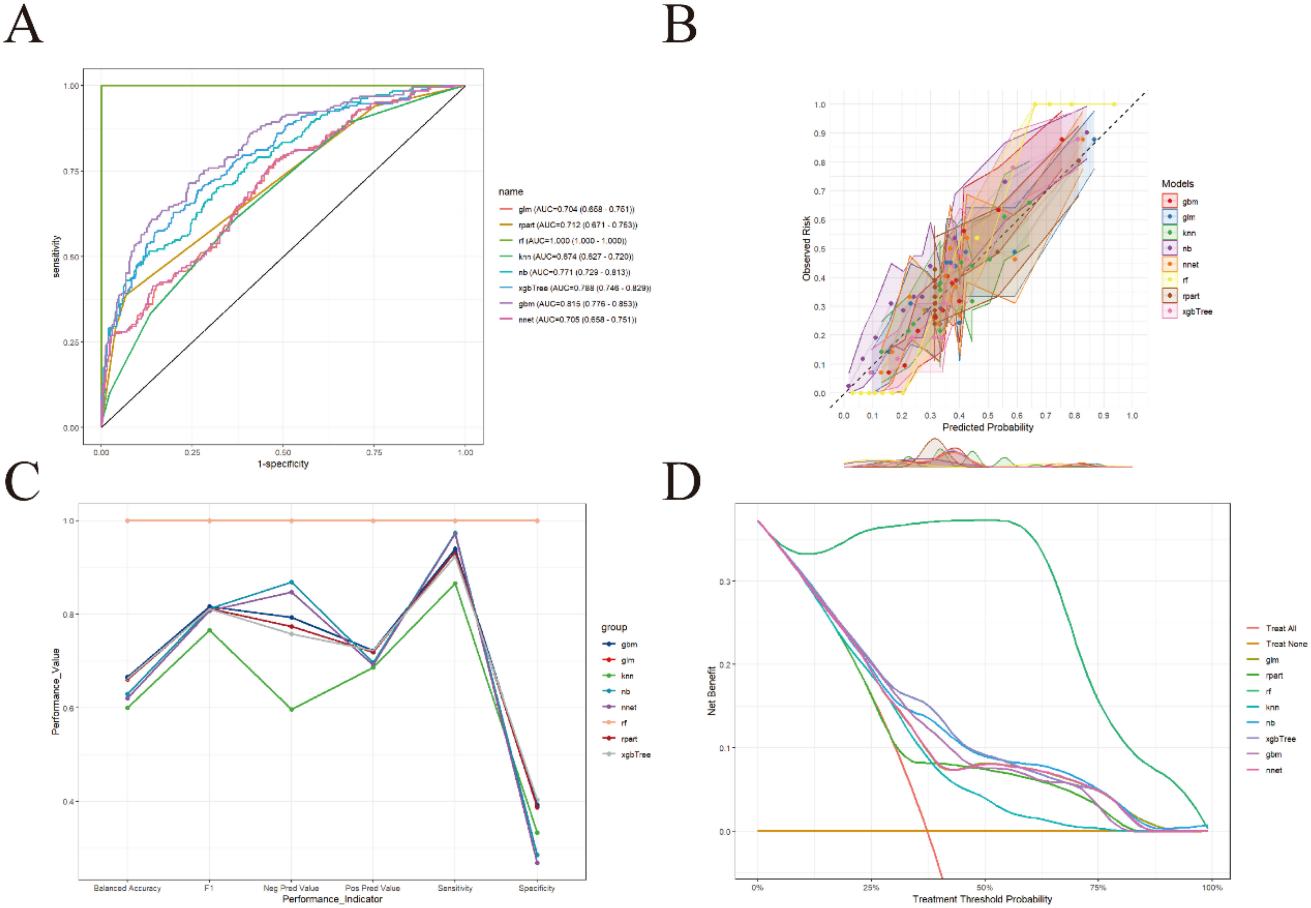
Performance of machine learning (ML) models in predicting lymph node metastasis (LNM) in colorectal cancer (CRC) patients in the training set. **(A)** ROC curve analysis **(B)**, calibration curve analysis **(C)**, parallel line graph of the evaluation metrics for each model **(D)**, and DCA curves for each model. glm (logistic regression), rpart (decision tree), rf (random forest), nb (naive Bayes), knn (K-nearest neighbor), xgbTree (extreme gradient boosting), gbm (stochastic gradient boosting), and nnet (neural network).

**Figure 3A** displays the receiver operating characteristic (ROC) curves for the training set. Within this cohort, the random forest (RF) model exhibited exceptional discrimination and calibration capabilities, achieving an AUC of 1.0 (95% CI: 1–1). The RF model also recorded the lowest Brier score (0.030) among all candidates, suggesting an excellent fit to the training data and robust predictive accuracy (**Table 3**). Calibration plots for all ML models in the training set (**Figure 3B**) further confirm the strong alignment and precision of the RF model. **Figure 3C** presents a parallel coordinate plot evaluating metrics across models. The RF model attained perfect scores (1.0) for balanced accuracy, F1 score, negative predictive value, positive predictive value, sensitivity, and specificity. The gradient boosting machine (GBM) model had an AUC of 0.815 (95% CI: 0.776–0.853) and a Brier score of 0.173. For the GBM model, the balanced accuracy was 0.666, the F1 score was 0.817, the negative predictive value was 0.793, the positive predictive value was 0.722, and the sensitivity reached 0.940. The specificity of the GBM model (0.392) is illustrated in **Figure 2C**. Decision curve analysis (DCA) demonstrated that both the RF model (threshold range: 0–1) and the GBM model (threshold range: 0–0.9) exhibited superior net benefit across the full spectrum of threshold probabilities (**Figure 3D**).

**Table 3:** The Brier scores of the machine learning models.

Dataset	glm	rpart	rf	knn	nb	xgbTree	gbm	nnet
Brier Sore Train	0.1947766	0.1903377	0.03032412	0.2121589	0.1865728	0.1797811	0.172516	0.1953456
Brier Sore Test	0.1906187	0.2018619	0.2119464	0.2371917	0.21174	0.1928701	0.195839	0.1903877

Note: glm (logistic regression), rpart (decision tree), rf (random forest), nb (naive Bayes), xgbTree (extreme gradient boosting), gbm (stochastic gradient boosting) and nnet (neural network).

**Figure 4:**
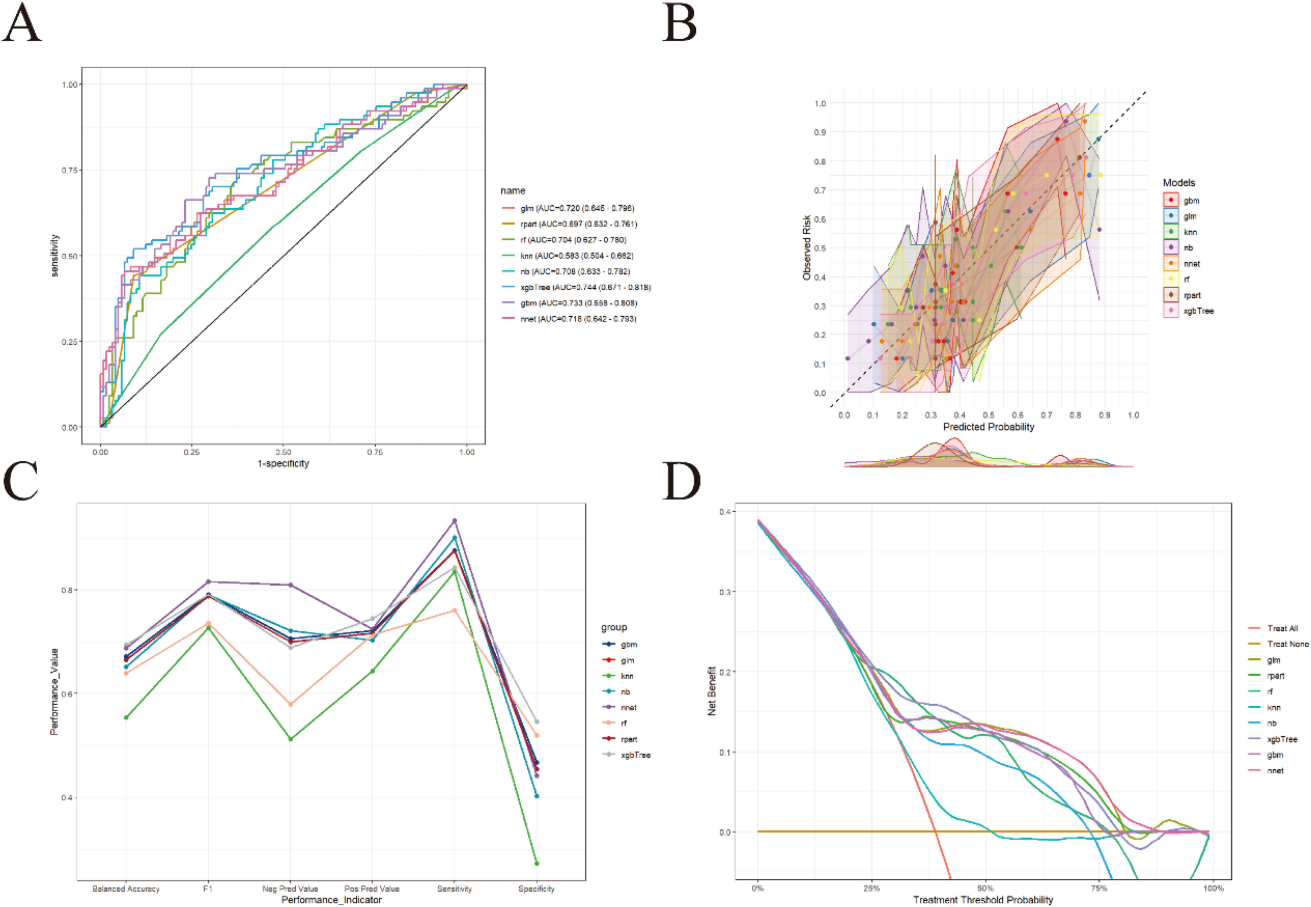
Performance of machine learning (ML) models in predicting lymph node metastasis (LNM) in colorectal cancer (CRC) patients in the test set. **(A)** ROC curve analysis **(B)**, calibration curve analysis **(C)**, parallel line graph of the evaluation metrics for each model **(D)** DCA curves for each model. glm (logistic regression), rpart (decision tree), rf (random forest), nb (naive Bayes), knn (K-nearest neighbor), xgbTree (extreme gradient boosting), gbm (stochastic gradient boosting), and nnet (neural network).

In the validation cohort, the XGBoost (xgbTree) algorithm achieved optimal performance regarding discrimination and calibration, yielding an area under the receiver operating characteristic curve (AUC) of 0.744 (95% confidence interval [CI]: 0.671–0.818; **Figure 4A**). The model attained a Brier score of 0.192, reflecting excellent goodness-of-fit to the training data and robust predictive precision (**Table 3**). Calibration plots for all machine learning algorithms in the test set are presented in **Figure 4B**, further confirming that the XGBoost model aligns closely with observed outcomes and maintains high accuracy. **Figure 4C** displays a parallel coordinate plot summarizing evaluation metrics for each algorithm within the validation cohort. Specifically, for the xgbTree model, balanced accuracy (0.694), F1 score (0.791), negative predictive value (0.689), positive predictive value (0.745), sensitivity (0.843), and specificity (0.545) are detailed in **Figure 4C**. Furthermore, DCA indicated that the xgbTree model (effective threshold range: 0–0.76) and the neural network (nnet) model (effective threshold range: 0–0.80) provided the greatest clinical utility across the entire range of threshold probabilities (**Figure 4D**).

Collectively, both the GBM and xgbTree models demonstrated strong efficacy in predicting lymph node metastasis (LNM) risk in colorectal cancer (CRC) patients across both training and validation datasets. To enhance model transparency and validate explainability, we derived feature importance rankings for the two best−performing models (gbm and xgbTree) using the built−in importance functions. For the gbm model, extramural vascular invasion (EMVI) and T stage were consistently the top two contributors to lymph node metastasis prediction, followed by fibrinogen (FIB), systolic blood pressure (SBP), thrombin time (TT), and alpha−fucosidase (AFU). The xgbTree model showed a similar pattern. Notably, these six variables were exactly those identified as independent predictors in the multivariate logistic regression analysis (**Table 2**). This concordance between the machine learning feature importance and conventional regression results enhances the interpretability of our models and confirms that the predictions are driven by clinically relevant factors rather than spurious correlations. The GBM model emerged as the primary choice for LNM risk stratification in CRC, with the xgbTree model serving as a close secondary option.

## 4 Discussion

### 4.1 Interpretation

This investigation successfully identified independent risk factors associated with LNM. Subsequently, we constructed and validated predictive machine learning models incorporating eight distinct algorithms alongside these identified risk factors to estimate LNM probability in CRC patients. Following comparative assessment, the GBM model was determined to be the most effective tool for predicting LNM risk, followed closely by the xgbTree model.

This model is intended for the preoperative prediction of lymph node metastasis in colorectal cancer. By using only preoperative variables, it can inform clinical decisions such as the need for neoadjuvant chemotherapy, the choice of surgical approach, or the extent of lymph node dissection before the patient undergoes any invasive procedure.

Multivariate logistic regression analysis performed on the training cohort identified six variables independently correlated with LNM risk in CRC: extramural vascular invasion (EMVI), clinical T stage, fibrinogen (FIB) levels, systolic blood pressure (SBP), tumor thickness (TT), and alpha-L-fucosidase (AFU). The literature consistently links EMVI with LNM. For instance, Ryan et al. reported EMVI as an independent predictor of LNM in mismatch repair-deficient (dMMR) CRC cases (odds ratio [OR] = 3.81; 95% CI: 1.56–9.19; P < 0.001) (34). Similarly, Abe et al. highlighted MRI-detected EMVI as a valuable imaging biomarker for identifying lateral lymph node metastasis in rectal cancer patients (35). Additionally, prior research has established EMVI as an independent determinant of pathological nodal status (OR = 3.476; 95% CI: 2.186–5.527; P < 0.001) (36). Our multivariate analysis corroborates these findings.

Furthermore, our results indicate that CRC patients presenting with advanced clinical T stages exhibit a heightened susceptibility to lymph node metastasis. Fu et al. previously documented a significant association between CT-assessed T stage and LN metastasis in CRC (P < 0.001) (37). Relative to T1 disease, the odds of LNM increased substantially for higher stages: 1.18-fold for T2 (OR = 1.18; 95% CI: 1.68–2.03), 5.40-fold for T3 (OR = 5.40; 95% CI: 4.96–5.89), and 8.89-fold for T4 (OR = 8.89; 95% CI: 8.08–9.80) (38). Mo et al. also identified T stage as an independent predictor of LNM status specifically in T1–T2 CRC patients (39). Notably, our study identifies elevated fibrinogen (FIB) levels as an independent risk factor for LNM. While direct correlations between serum FIB concentrations and LNM in CRC have been infrequently reported in previous literature, Wen et al. demonstrated that the fibrinogen-to-albumin ratio (FAR) serves as an independent predictor of lymph node metastasis (LNM) in colorectal cancer (CRC), with an odds ratio of 1.145 (95% CI: 1.062–1.235; P < 0.001) (40). Similarly, Liu and colleagues identified a significant inverse relationship between elevated fibrinogen-to-prealbumin ratios (FPR) and overall survival (OS) in CRC cohorts, establishing FPR as an independent prognostic marker (HR = 2.669, 95% CI: 1.052–6.772) (41). Furthermore, circulating fibrinogen levels correlate positively with tumor recurrence, T stage, and TNM classification. Notably, hyperfibrinogenemia often precedes systemic inflammatory responses yet remains clinically associated with recurrent disease (42). Mechanistically, fibrinogen acts as a molecular bridge linking tumor cells, platelets, and vascular endothelium, thereby enhancing intercellular adhesion and facilitating metastatic dissemination (43). Consequently, we hypothesize that elevated fibrinogen concentrations may promote LNM via this adhesive mechanism, although dedicated investigations are required to elucidate the specific FIB-LNM axis in CRC.

Our analysis further identified systolic blood pressure (SBP) as an independent risk factor for LNM in CRC patients. While no prior literature directly links SBP to LNM in this context, existing evidence indicates that renin-angiotensin system (RAS) components, including angiotensin, function as growth factors driving cellular proliferation and angiogenesis; their dysregulation correlates with adverse outcomes in CRC (44). Given the central role of RAS in blood pressure homeostasis, we postulate that aberrant RAS signaling may concurrently influence both hemodynamic parameters and metastatic potential. Nevertheless, prospective studies are necessary to validate this conjecture.

Additionally, thrombin time (TT) emerged as an independent predictor of LNM risk. Although direct associations between TT and LNM remain underexplored, Hanly et al. reported that thrombomodulin (TM), an endothelial receptor exhibiting anticoagulant properties through thrombin inhibition and modulation of cellular adhesion, shows reduced expression in advanced-stage malignancies (45). Since neoplasms profoundly perturb hemostatic equilibrium—where coagulation factors, anticoagulants, activators, and fibrinolytic inhibitors collectively shape tumor biology (45)—we speculate that altered TT values may reflect underlying clotting system activation or suppression, thereby influencing tumor progression and distant spread. Further inquiry is warranted to confirm this hypothesis.

Finally, our findings highlight alpha-L-fucosidase (AFU) levels as a significant factor associated with LNM susceptibility. Previous reports indicate significantly diminished AFU enzymatic activity in CRC patients compared to healthy controls (P < 0.001) (46), while other studies suggest that AFU activity serves as an independent prognostic indicator for tumor recurrence (47). Moreover, combining CD26 with AFU yields high diagnostic sensitivity and specificity for CRC detection (48). To our knowledge, this investigation represents an inaugural report linking AFU concentration to LNM risk in CRC populations.

To rigorously assess the predictive efficacy of our machine learning models, we employed a comprehensive suite of goodness-of-fit metrics, including area under the receiver operating characteristic curve (AUC-ROC), calibration plots, balanced accuracy, F1 scores, and decision curve analysis (DCA).

To rigorously assess the predictive efficacy of our machine learning (ML) algorithms, we conducted comprehensive evaluations focusing on both calibration and discriminative capacity. It is noteworthy that the validation and test cohorts were sourced from distinct medical institutions; this heterogeneity likely accounts for the observed performance discrepancies between these two sets. Furthermore, the random forest (RF) model exhibited an area under the curve (AUC) of 1.0 on the training data, a finding indicative of overfitting. The perfect performance of the random forest (RF) model in the training set (AUC = 1.0, sensitivity = 1.0, specificity = 1.0) warrants careful discussion. This finding is a classic indicator of overfitting, a phenomenon where a model learns not only the underlying patterns but also the noise specific to the training data, leading to grossly optimistic performance estimates that do not generalize to new data (42). Several factors likely contributed to overfitting in our RF model. First, although RF is generally robust to overfitting due to its bagging (bootstrap aggregating) mechanism and random feature selection, it remains vulnerable when the training sample size is moderate (n = 499) relative to the number of candidate predictors (over 40 variables). Second, the signal−to−noise ratio in medical datasets is often limited, and RF can exploit spurious correlations that exist by chance in the training set, especially when no regularization or early stopping is applied. Third, while we used ten−fold cross−validation for hyperparameter tuning (e.g., tuning mtry), the final reported training performance was evaluated on the same training set without cross−validation, which can produce upward−biased estimates. Importantly, because of this clear overfitting signal, we did not select RF as our final model. Instead, we chose the gradient boosting machine (gbm) and xgbTree models, which demonstrated strong but not perfect training performance (AUC 0.815 and 0.813, respectively) and, more critically, maintained reasonable discriminative ability on the external validation set (AUC 0.733 and 0.744, respectively). The decision to prioritize these models over RF was based on their superior generalizability, as reflected by the smaller drop in AUC from training to external validation. Consequently, we relied on internal validation metrics to derive more realistic estimates of model generalizability. Within this internal validation framework, both the gradient boosting machine (gbm) and extreme gradient boosting (xgbTree) models demonstrated robust performance in forecasting the risk of LNM in colorectal cancer (CRC). The consistency of evaluation outcomes across diverse datasets further substantiates the model’s robustness. Nevertheless, our conclusions may be subject to bias stemming from the retrospective nature of the study and limited sample sizes. To bolster the assessment of model stability and generalizability, future research will prioritize prospective studies incorporating additional external validation cohorts.

Previously, numerous investigations constructed predictive models for LNM in CRC patients. For instance, Kang et al. employed a least absolute shrinkage and selection operator (LASSO)-based ML algorithm to predict LNM in T1 CRC cases. Their LASSO-ML model, which integrated histopathological parameters and tumor-infiltrating lymphocytes (TILs), outperformed conventional Japanese criteria (49). Similarly, Lei et al. retrospectively analyzed 300 CRC patients treated at two Peking University Shenzhen hospitals throughout 2021, developing a potent risk prediction model for LNM based on ML-derived features extracted from primary tumor histology and clinicopathological variables (49). Additionally, other researchers have established supervised learning models utilizing histopathological metrics and inflammatory markers to predict LNM status in pT1NxM0 CRC patients (50). However, a predominant limitation of these prior studies is their reliance on histopathological parameters, which are often costly and difficult to acquire. In contrast, the predictive framework developed in this study incorporates variables that are readily accessible and evaluable during a patient’s hospitalization, thereby offering a practical tool for effective LNM risk stratification. A further strength of our work lies in the comparative analysis of various ML algorithms, which facilitated the selection of the optimal model. Evaluations using external validation sets confirmed that the selected model possesses strong generalizability.

### 4.2 Limitations

Several limitations of this study warrant acknowledgment. First, the retrospective design inherently introduces potential biases that are difficult to eliminate entirely. Second, due to constraints associated with retrospective data collection, certain clinicopathological factors that might influence lymph node metastasis (LNM) were not included in our analysis. Third, the sample size, while comparable to or larger than many published machine learning studies in colorectal cancer, remains relatively modest for developing and validating complex ML algorithms. With 499 patients in the training cohort and 198 in the external validation cohort, there is a risk that model performance estimates may be unstable, and some predictors with small effect sizes could be missed. This concern is particularly relevant for algorithms with many tunable parameters, such as random forest, gradient boosting, and neural networks. To mitigate this, we employed ten−fold cross−validation (repeated across multiple iterations) to maximize data efficiency and reduce overfitting. We also prioritized external validation over purely internal validation, as the former provides a more realistic assessment of generalizability. Nevertheless, a larger sample size would likely improve model stability, narrow confidence intervals around performance metrics, and potentially identify additional predictors. Fourth, and importantly, our model was developed exclusively using data from Chinese patients recruited from two tertiary referral hospitals in Hebei Province. As highlighted by the reviewer, the quality and representativeness of training data are critical to ensure model fairness. Although we applied rigorous exclusion criteria and external validation, the retrospective design and single−ethnicity (Chinese) population may introduce selection bias and limit generalizability to other racial, ethnic, or socioeconomic groups, as well as to different geographic regions or healthcare systems. Factors such as genetic background, environmental exposures, dietary patterns, and screening practices may differ across populations and could influence LNM risk and model performance. Therefore, our model should be interpreted as a proof−of−concept requiring rigorous external validation in diverse populations before any clinical implementation can be recommended. Despite these constraints, our finalized predictive framework demonstrated robust efficacy across both internal cross−validation and external validation phases. Future investigations will focus on expanding the sample size, implementing data balancing strategies, and prioritizing prospective, multicenter, and diverse−population cohorts to further enhance the model’s generalizability and predictive precision. Additionally, techniques such as data pooling across institutions or the use of transfer learning could be explored to overcome sample size limitations in future work.

### 4.3 Conclusions

In summary, this research successfully constructed and validated machine learning algorithms designed to forecast the likelihood of LNM in individuals with CRC. Both the Gradient Boosting Machine (gbm) and the Extreme Gradient Boosting (xgbTree) models exhibited superior predictive capabilities for CRC-related LNM within both the training and testing datasets. These computational tools offer significant utility for clinicians in accurately identifying LNM status among CRC patients, thereby establishing a foundational basis for formulating personalized therapeutic regimens.

## Data Availability

The raw data supporting the conclusions of this article will be made available by the authors, without undue reservation.
