# Supplementary material for: Machine learning to develop and validate a model for predicting the risk of lymph node metastasis in colorectal cancer patients
Source: Front Oncol. 2026 Jul 21;16:1860520. doi: 10.3389/fonc.2026.1860520 (PMC13433173; doi:10.3389/fonc.2026.1860520)
Supplement: Supplementary file 1 [file Table1.docx]

| Algorithm | Hyperparameter | Tuning grid | Final optimal value |
| --- | --- | --- | --- |
| Logistic Regression (LR) | none | — | — |
| Decision Tree (DT) | cp (complexity parameter) | 0.0001, 0.001, 0.01, 0.05, 0.1 | 0.01 |
| Random Forest (RF) | mtry | 2, 4, 6, 8, 10 | 4 |
| K-Nearest Neighbors (KNN) | k | 1, 3, 5, 7, 9, 11, 13 | 7 |
| Naive Bayes (NB) | fL (Laplace correction) | 0, 0.5, 1 | 0 |
|  | usekernel | TRUE, FALSE | TRUE |
| Extreme Gradient Boosting (xgbTree) | nrounds | 50, 100, 150 | 100 |
|  | max_depth | 3, 4, 5, 6 | 4 |
|  | eta | 0.01, 0.05, 0.1, 0.3 | 0.1 |
|  | gamma | 0, 0.1, 0.2 | 0 |
|  | colsample_bytree | 0.6, 0.8, 1.0 | 0.8 |
|  | min_child_weight | 1, 3, 5 | 1 |
|  | subsample | 0.5, 0.75, 1.0 | 0.75 |
| Stochastic Gradient Boosting (gbm) | n.trees | 50, 100, 150, 200 | 150 |
|  | interaction.depth | 1, 2, 3, 4 | 3 |
|  | shrinkage | 0.01, 0.05, 0.1, 0.2 | 0.1 |
|  | n.minobsinnode | 5, 10, 15 | 10 |
| Neural Network (nnet) | size | 1, 3, 5, 7 | 3 |
|  | decay | 0, 0.1, 0.01, 0.001 | 0.01 |

Supplementary Table S1. Hyperparameter tuning grids and final optimal values for each machine learning algorithm.
